# Large-scale analysis of brain-wide electrophysiological diversity reveals novel characterization of mammalian neuron types

**DOI:** 10.1186/1471-2202-16-S1-O4

**Published:** 2015-12-18

**Authors:** Shreejoy J Tripathy, Dmitry Tebaykin, Brenna Li, Ogan Marcarci, Lilah Toker, Paul Pavlidis

**Affiliations:** 1 Centre for High-Throughput Biology, University of British Columbia, Vancouver, BC V6K 2B7, Canada

## 

Brains achieve efficient function through implementing a division of labor, in which different neurons serve distinct computational roles. One striking way in which neuron types differ is in their electrophysiology properties. These properties arise through expression of combinations of ion channels that collectively define the computations that a neuron performs on its inputs and its role within its larger circuit. Though the electrophysiology of many neuron types has been previously characterized, these data exist across thousands of journal articles, making cross-study neuron-to-neuron comparisons difficult.

**Figure 1:**
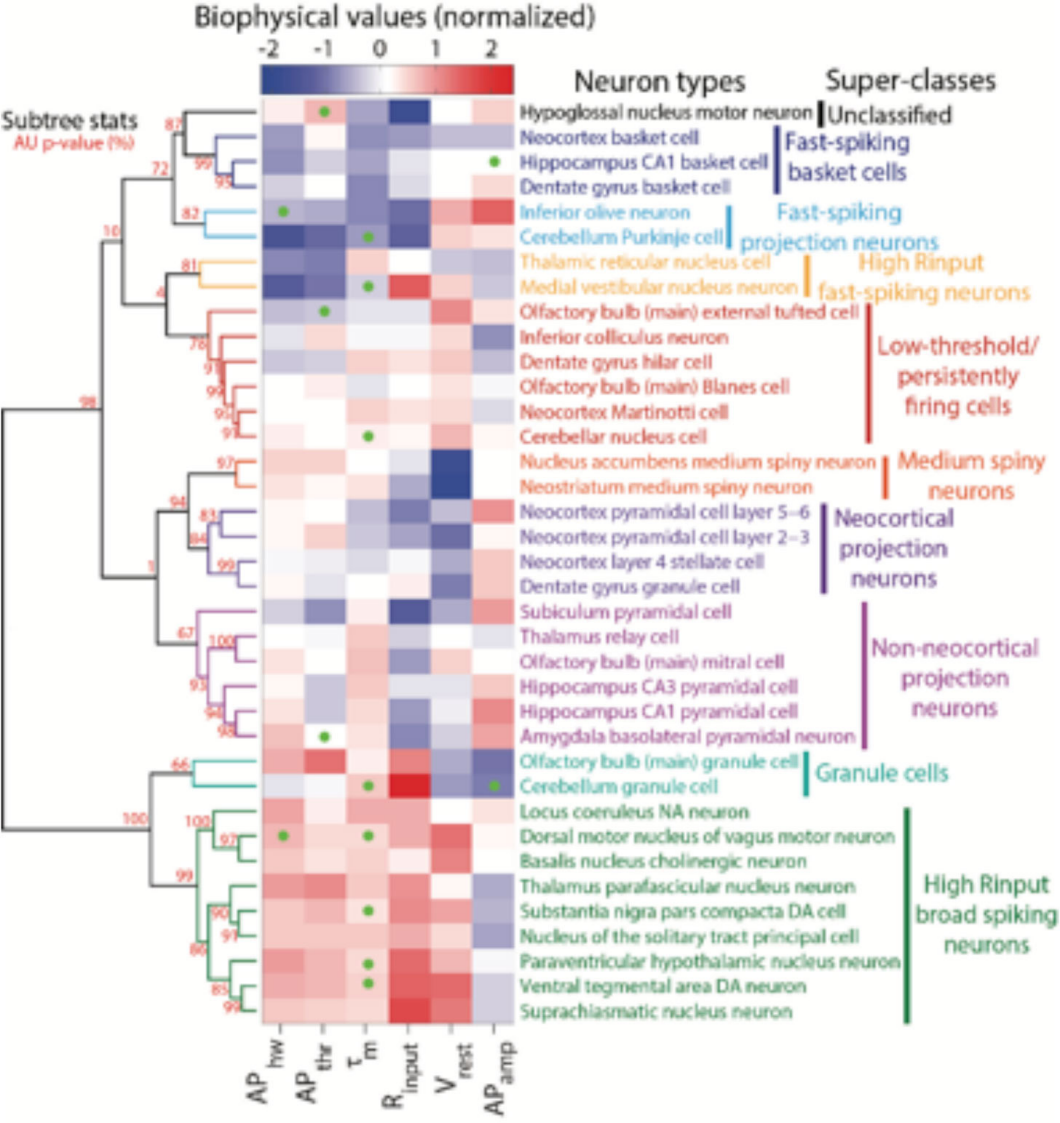
**Hierarchical clustering of diverse neuron types on the basis of electrophysiological similarity**. Electrophysiological parameters are obtained from the NeuroElectro database via literature-mining and are normalized to account for variability in experimental methodologies across studies.

Here, we present NeuroElectro, a public database where physiological properties for the majority of mammalian neuron types have been compiled through semi-automated literature text-mining and expert curation. The corresponding web application, at http://www.neuroelectro.org, provides a rich dynamic interface for visualizing and comparing physiological information across neuron types; conveniently linking extracted data back to its primary reference. Mining the database content after normalization for methodological differences, we show that there exist but 5-9 major neuron classes in terms of electrophysiological properties, which separate largely based on cell size and basal levels of excitability (Figure 1).

As an example of how this resource can help answer fundamental questions in neuroscience, we integrate NeuroElectro with neuronal gene expression from public datasets like the Allen Brain Atlas. We show that simple statistical models can accurately predict features of a neuron's electrophysiological phenotype given information of its gene expression alone. We further investigate these models to ask which genes, of the 20K in the genome, are most predictive of neuron physiology. We find that while ion channel-related genes provide significant predictive power, the most predictive gene classes surprisingly correspond to G-proteins and transcription factors, suggesting the involvement of hundreds of diverse genes in regulating a neuron's computational function.

